# Practical considerations in an era of multicolor optogenetics

**DOI:** 10.3389/fncel.2023.1160245

**Published:** 2023-05-24

**Authors:** Daniel J. Rindner, Gyorgy Lur

**Affiliations:** Department of Neurobiology and Behavior, University of California, Irvine, Irvine, CA, United States

**Keywords:** optogenetics, crosstalk, dual-color, red-shifted, channelrhodopsin, stimulation

## Abstract

The ability to control synaptic communication is indispensable to modern neuroscience. Until recently, only single-pathway manipulations were possible due to limited availability of opsins activated by distinct wavelengths. However, extensive protein engineering and screening efforts have drastically expanded the optogenetic toolkit, ushering in an era of multicolor approaches for studying neural circuits. Nonetheless, opsins with truly discrete spectra are scarce. Experimenters must therefore take care to avoid unintended cross-activation of optogenetic tools (crosstalk). Here, we demonstrate the multidimensional nature of crosstalk in a single model synaptic pathway, testing stimulus wavelength, irradiance, duration, and opsin choice. We then propose a “lookup table” method for maximizing the dynamic range of opsin responses on an experiment-by-experiment basis.

## Introduction

**FIGURE 1:**
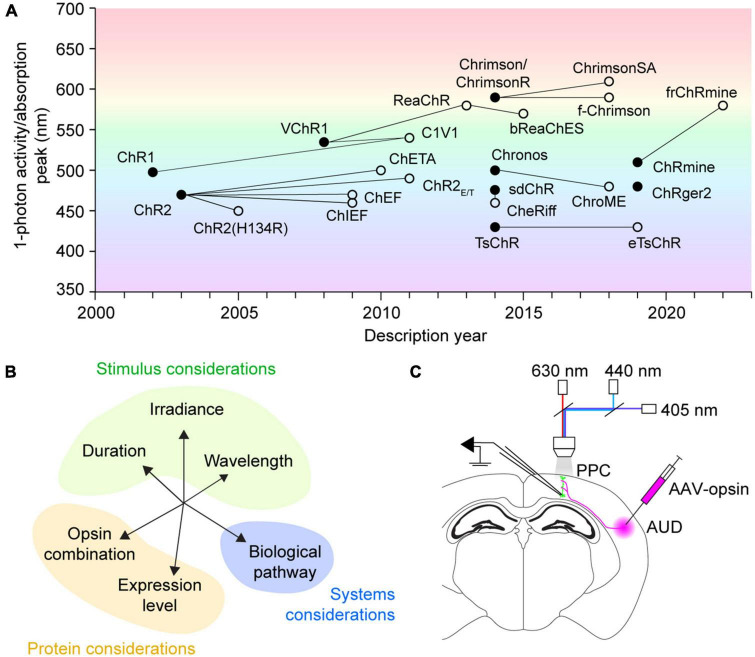
History, concerns, and testing of multicolor optogenetics. **(A)** Discovery history of commonly used channelrhodopsins for neural excitation. Filled circles designate novel opsins discovered by screening or created with rational design. Empty circles indicate mutant opsin variants, with connecting lines to their protein backbones. The mutant variant ChrimsonR overlaps with the native opsin Chrimson. Figure background color illustrates the wavelength of 1-photon activity/absorption peaks. **(B)** A representation of the multidimensional factors affecting opsin crosstalk. Each should be considered when designing any multicolor optogenetic experiment. **(C)** Schematic of experimental testing for crosstalk between excitatory optogenetic actuators for use in acute slice synapse activation applications. PPC, posterior parietal cortex; AUD, auditory cortex; AAV-opsin, adeno-associated virus carrying opsin construct, either ChR2(H134R), Chronos, or ChrimsonR.

Multicolor optogenetic approaches are enormously valuable for studying the function of complex neural circuits. Optogenetic constructs with distinct wavelength sensitivity can be combined in actuator pairs, sensor pairs, or an actuator and sensor together in independent pathways, relying on spectral separation to bypass limitations imposed by spatial overlap of the tools. However, most current red-shifted optogenetic constructs exhibit sensitivity to blue light, leading to cross-activation concerns regardless of the precise tool combination used. For our exploration of crosstalk, we focus exclusively on excitatory actuators. Optogenetic actuators include light-activated ion channels, ion pumps, and G protein-coupled receptors (Rost et al., 2017; Emiliani et al., 2022), of which channelrhodopsins are perhaps the most widely used for circuit manipulation. The earliest described channelrhodopsins were channelrhodopsin-1 (ChR1) (Nagel et al., 2002) and channelrhodopsin-2 (ChR2) (Nagel et al., 2003), discovered in the algal species *C. reinhardtii* (Figure 1A). ChR2 quickly became the protein backbone of many engineering efforts to increase speed (Lin et al., 2009; Gunaydin et al., 2010) and photocurrent amplitude (Nagel et al., 2005; Berndt et al., 2011), leading to the optimization of the protein for mammalian expression (Boyden et al., 2005). The excitation peak of wild-type ChR2 is at 470 nm (Nagel et al., 2003). Similarly, activation spectra for many popular mutant variants–ChR2(H134R) (Nagel et al., 2005), ChEF/ChIEF (Lin et al., 2009), ChETA (Gunaydin et al., 2010), ChR2(E123T/T159C) (Berndt et al., 2011)–as well as newly identified channelrhodopsins such as sdChR (Hochbaum et al., 2014) and Chronos (Klapoetke et al., 2014), peak in the 460–500 nm range (Figure 1A). Notably, these blue-light-activated opsins [referred to as “blue opsin(s)” going forward] also exhibit minimal sensitivity to wavelengths above 550 nm. Thus, an ideal red-shifted actuator for dual-color applications will be strongly activated by wavelengths longer than 550 nm, and insensitive to those under 500 nm.

VChR1, discovered in the algae *V. carteri*, was the first-reported opsin red-shifted over 50 nm from ChR2, with an excitation peak at 530 nm (Zhang et al., 2008; Figure 1A). However, VChR1 has considerable blue-light sensitivity, which prevented its immediate use in dual-color applications. Perhaps the first widely adopted red opsin was the fusion protein C1V1 (Yizhar et al., 2011), a portmanteau of its component opsins ChR1 and VChR1. While the C1V1 peak absorption was hardly red-shifted compared to VChR1, it had appreciably less sensitivity to wavelengths under 500 nm (Yizhar et al., 2011). Still, C1V1 retains ∼40% absorbance of 470 nm light commonly used for activating blue optogenetic tools (Yizhar et al., 2011). The search for other red-shifted channelrhodopsins has yielded many protein alternatives, including ReaChR (Lin et al., 2013), Chrimson and ChrimsonR (Klapoetke et al., 2014), ChrimsonSA (Oda et al., 2018), ChRmine (Marshel et al., 2019), and frChRmine (Kishi et al., 2022). Nonetheless, all currently known red-shifted actuators [referred to as “red opsin(s)” going forward] exhibit non-negligible blue-light sensitivity, which can lead to possible cross-activation during blue stimulation periods if not properly controlled for.

Two strategies exist to minimize crosstalk, which have been applied to opsin combinations including Chronos/ChrimsonR (Klapoetke et al., 2014; Bauer et al., 2021; Christoffel et al., 2021), CheRiff/ChrimsonR (Anisimova et al., 2023), ChR2/ReaChR (Hooks et al., 2015), and ChR2(H134R)/ChrimsonR (Chiu et al., 2018; Birdsong et al., 2019; Prasad et al., 2020; Xia et al., 2020; Joffe et al., 2022; Rindner et al., 2022). Indeed, the precise strategy used will depend on experimental context. The first, introduced by Hooks et al. (2015), is often used to test pathway convergence (Prasad et al., 2020; Bauer et al., 2021; Shelton et al., 2022). This approach involves utilizing a long, 50–250 ms, red light stimulus to forcibly inactivate the red opsin expressing neuron population before immediately stimulating the blue opsin. As the blue stimulus is applied while axons expressing the red opsin remain unresponsive, any postsynaptic response can be solely attributed to activation of blue opsin expressing cells. However, this method is not feasible in contexts where precise timing between blue and red channels is desired, for example in studies of spike-timing-dependent plasticity (Anisimova et al., 2023) or synaptic integration (Xia et al., 2020; Rindner et al., 2022). A second strategy is to limit stimulation parameters to ranges which do not cross-activate opsins (Klapoetke et al., 2014; Birdsong et al., 2019; Xia et al., 2020; Joffe et al., 2022; Rindner et al., 2022; Anisimova et al., 2023). With this approach, blue stimulus irradiance, and less commonly duration, are first titrated in a separate experimental population where only the red opsin is expressed. The upper blue light exposure limit averting red opsin cross-activation is then used across all later experiments. This approach requires thorough testing (described in detail in Section “Results”), however, it allows maximum temporal control of independent neuron populations. A caveat of this approach is that population-derived blue light exposure limits may provide inadequate excitation in instances where blue opsin expression levels are low, resulting in cases where experiments must be discarded (Xia et al., 2020). This is particularly true when near-violet stimulation wavelengths, over 50 nm blue-shifted from the excitation peak of most blue opsins, are chosen [for example, 405 nm in Anisimova et al. (2023)] in an effort to minimize cross-activation of red opsins. Thus, alternative strategies that maximize the dynamic range of crosstalk-free blue stimulus could improve the throughput of dual-color optogenetic experiments.

Here, we focus on a single synaptic pathway to test how the scope of experimental variables chosen by the investigator (Figure 1B) influences crosstalk risk, showcasing an example control experiment expanding on those done by others. We demonstrate an exhaustive test of crosstalk parameters, systematically varying the irradiance and duration of three different stimulus wavelengths–405, 440, and 630 nm. In consideration of the range of opsins available, we furthermore test crosstalk between three commonly used actuators–ChR2(H134R), Chronos, and ChrimsonR. We lastly propose a “lookup table” approach leveraging red opsin responses on a cell-by-cell basis to maximize crosstalk-free blue excitation in multicolor optogenetic experiments.

## Results

### Multidimensional considerations in crosstalk testing

**FIGURE 2:**
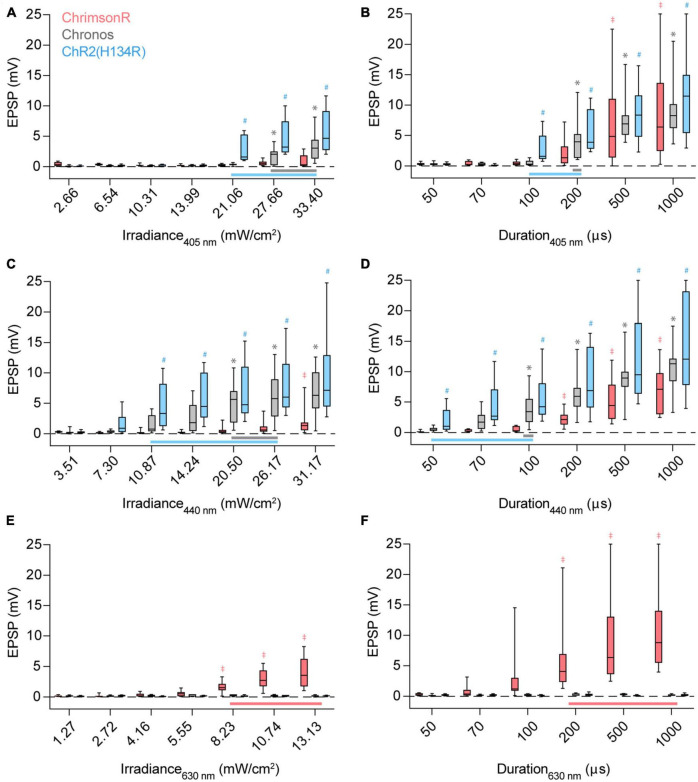
Stimulus irradiance, duration, wavelength, and opsin choice contribute to crosstalk risk. **(A)** Excitatory postsynaptic potential (EPSP) amplitudes when ChrimsonR (red), Chronos (gray), or ChR2(H134R) (blue)-expressing afferents are stimulated by a 100 μs pulse of 405 nm light with increasing irradiance. Vertical bars extend from the 25th to 75th percentiles, with the median represented by a horizontal line. Whiskers indicate minimum and maximum recorded values. Horizontal bars at bottom **(A–D)** represent the stimulus parameter range which can be used to activate Chronos (gray) or ChR2(H134R) (blue) without cross-activating ChrimsonR. Symbols above the boxes indicate opsin [*: Chronos, ^#^: ChR2(H134R), ^‡^: ChrimsonR] and stimulus parameter combinations resulting in population-level non-zero responses [*p* < 0.05, *t*-test, experimental replicates (*n*) listed in Table 1]. **(B)** Response amplitudes when afferents are stimulated with a 21.06 mW/cm^2^ pulse of 405 nm light with increasing pulse durations. **(C)** Response amplitudes when afferents are stimulated with a 100 μs pulse of 440 nm light with increasing irradiance. **(D)** Response amplitudes when afferents are stimulated with a 20.50 mW/cm^2^ pulse of 440 nm light with increasing pulse durations. **(E)** Response amplitudes when afferents are stimulated with a 100 μs pulse of 630 nm light with increasing irradiance. **(F)** Response amplitudes when afferents are stimulated with a 8.23 mW/cm^2^ pulse of 630 nm light with increasing pulse durations. Red horizontal bar at bottom **(E,F)** represents the stimulus parameter range which can be used to activate ChrimsonR without cross-activating Chronos and ChR2(H134R). ^‡,*,#^*p* < 0.05, *t*-test.

To demonstrate how stimulus wavelength, irradiance, duration, and opsin choice affect crosstalk in a system, we first chose a model synaptic pathway from the auditory cortex (AUD) to the posterior parietal cortex (PPC). AUD neurons synapse on pyramidal cells in PPC layer 5 and provide strong excitation when optogenetically stimulated (Rindner et al., 2022). We expressed ChR2(H134R), Chronos, or ChrimsonR in AUD using adeno-associated viral vectors (see Section “Methods”) and recorded light-evoked responses in layer 5 pyramidal cells of the PPC using whole-cell patch-clamp (Figure 1C). By varying stimulus irradiance or duration, we determined the dose-response relationship to excitation by 405 nm (blue/violet) (Figures 2A, B), 440 nm (blue) (Figures 2C, D), and 630 nm (red) (Figures 2E, F) LEDs. When expressed in AUD afferents, ChR2(H134R) and Chronos drove robust postsynaptic responses to 405 and 440 nm stimuli (Figures 2A–D). Stimulation of either opsin with 630 nm resulted in no detectable response (see Section “Methods”) across the range of irradiances or durations tested (Figures 2E, F; Table 1). In contrast, when afferents expressed ChrimsonR, light-evoked responses could be observed to stimulation with 405 nm (Figure 2B), 440 nm (Figures 2C, D) and 630 nm (Figures 2E, F; Table 1) wavelengths, as expected given the blue shoulder of the ChrimsonR activation spectra (Klapoetke et al., 2014). Notably, brief, low-irradiance stimulation at 405 nm and 440 nm averted ChrimsonR responses while eliciting EPSPs driven by ChR2(H134R)- and Chronos-expressing afferents (Figures 2A–D). This parameter range represents the ideal excitation window (indicated by horizontal bars in Figures 2A–D) where ChrimsonR can be used in tandem with ChR2(H134R) (light blue) or Chronos (gray) without substantial risk of cross-activation on a population level. To determine the ideal blue opsin and wavelength combination for high dynamic range, crosstalk-free use with ChrimsonR, we next collapsed across irradiance and duration and compared peak ChR2(H134R) and Chronos responses at the maximum 405 and 440 nm radiant exposure level averting ChrimsonR responses (405 nm: 42.12 mJ/m^2^; 440 nm: 26.17 mJ/m^2^). Largest crosstalk-free blue opsin evoked EPSPs were obtained when ChR2(H134R)-expressing afferents were stimulated at 440 nm (ChR2(H134R): 11.14 mV at 405 nm, 17.31 mV at 440 nm; Chronos: 12.09 mV at 405 nm, 13.05 mV at 440 nm). These results suggest that, within the tested blue opsin and wavelength combinations, ChR2(H134R) excited at 440 nm allowed for the highest dynamic range activation of the AUD to PPC synaptic pathway.

**TABLE 1:** Experimental replicates and statistical test results.

	405 nm irradiance (mW/cm^2^)							405 nm duration (μs)					
	2.66	6.54	10.31	13.99	21.06	27.66	30.4	50	70	100	200	500	1,000
ChrimsonR	(7)	(7)	(8)	(8)	(8)	(8)	(8)	(8)	(8)	(8)	(8)	(8)	(8)
	0.996	0.999	>0.999	>0.999	0.999	0.997	0.747	>0.999	>0.999	0.999	0.158	**0.03**	**0.023**
Chronos	(9)	(9)	(9)	(9)	(11)	(11)	(11)	(10)	(10)	(10)	(11)	(11)	(11)
	>0.999	>0.999	>0.999	>0.999	>0.999	**0.031**	**0.002**	>0.999	>0.999	0.976	**0.003**	**<0.001**	**<0.001**
ChR2(H134R)	(8)	(8)	(8)	(8)	(8)	(8)	(8)	(10)	(10)	(10)	(10)	(10)	(10)
	>0.999	>0.999	>0.999	0.999	**0.018**	**0.005**	**0.003**	>0.999	>0.999	**0.022**	**0.001**	**<0.001**	**<0.001**
	**440 nm irradiance (mW/cm^2^)**							**440 nm duration (μs)**					
	**3.51**	**7.3**	**10.87**	**14.24**	**20.5**	**26.17**	**31.17**	**50**	**70**	**100**	**200**	**500**	**1,000**
ChrimsonR	(11)	(11)	(11)	(11)	(11)	(11)	(11)	(11)	(11)	(11)	(11)	(11)	(11)
	>0.999	>0.999	>0.999	>0.999	0.887	0.209	**0.043**	>0.999	0.997	0.762	**<0.001**	**<0.001**	**<0.001**
Chronos	(10)	(10)	(10)	(10)	(11)	(11)	(11)	(12)	(12)	(12)	(12)	(12)	(12)
	>0.999	>0.999	0.058	0.054	**<0.001**	**<0.001**	**<0.001**	0.994	0.059	**0.002**	**<0.001**	**<0.001**	**<0.001**
ChR2(H134R)	(13)	(14)	(14)	(14)	(14)	(14)	(14)	(14)	(14)	(14)	(14)	(14)	(14)
	>0.999	0.361	**<0.001**	**<0.001**	**<0.001**	**<0.001**	**<0.001**	**0.017**	**<0.001**	**<0.001**	**<0.001**	**<0.001**	**<0.001**
	**630 nm irradiance (mW/cm^2^)**							**630 nm duration (μs)**					
	**1.27**	**2.72**	**4.16**	**5.55**	**8.23**	**10.74**	**13.13**	**50**	**70**	**100**	**200**	**500**	**1,000**
ChrimsonR	(11)	(11)	(11)	(11)	(12)	(12)	(12)	(11)	(11)	(11)	(11)	(11)	(11)
	>0.999	>0.999	>0.999	0.98	**0.001**	**<0.001**	**<0.001**	>0.999	0.62	0.069	**0.005**	**<0.001**	**<0.001**
Chronos	(7)	(7)	(7)	(7)	(7)	(7)	(7)	(7)	(7)	(7)	(7)	(8)	(8)
	>0.999	>0.999	>0.999	>0.999	0.996	0.998	>0.999	0.999	>0.999	>0.999	0.999	>0.999	>0.999
ChR2(H134R)	(8)	(8)	(8)	(8)	(8)	(8)	(8)	(9)	(9)	(9)	(9)	(9)	(9)
	>0.999	>0.999	>0.999	>0.999	0.992	>0.999	>0.999	>0.999	>0.999	0.991	0.998	>0.999	>0.999

Experimental replicates (n) (top) and paired *t*-test *p*-values (bottom) of the recorded postsynaptic response to opsin excitation by indicated stimulus parameters. Bolded *p*-values indicate *p* < 0.05.

### Experiment-to-experiment maximization of crosstalk-free blue excitation

**FIGURE 3:**
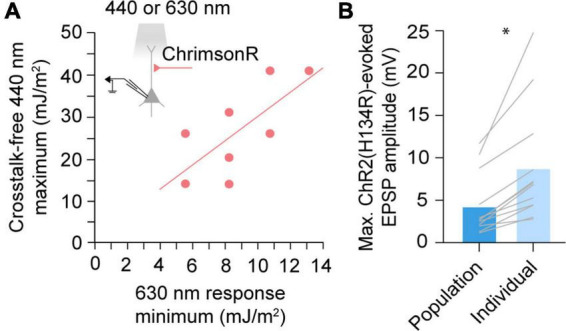
A red opsin “lookup table” for maximizing blue stimulus dynamic range. **(A)** Comparison of the minimum 630 nm radiant exposure necessary to elicit a detectable ChrimsonR postsynaptic response and maximum 440 nm radiant exposure averting a cross-activated excitatory postsynaptic potential (EPSP). Dots represent individual cells. Solid line represents the linear regression. Pearson *r* = 0.715, *n* = 8, *p* = 0.046. **(B)** Maximum evoked EPSP amplitudes when afferents expressing ChR2(H134R) are stimulated at population-derived or individually-derived blue radiant exposure limits. Gray lines represent individual cells. *p < 0.05, *n* = 12, paired *t*-test.

By definition, a population-derived maximum usable blue light exposure underestimates the true crosstalk-free exposure limit in a majority of experiments. To maximize crosstalk-free blue light stimulus, we next asked whether the upper limit of blue radiant exposure could be determined on an experiment-to-experiment basis, accounting for variable red opsin expression levels. We hypothesized that in dual-color experiments, red light responses could be used to estimate red opsin expression levels and predict sensitivity to blue light induced cross-activation. Indeed, within-cell comparison of EPSPs evoked by ChrimsonR-expressing afferents suggested that the maximum usable 440 nm radiant exposure level that averts cross-activation, increases linearly with the minimum 630 nm exposure necessary to evoke a response (Pearson *r* = 0.715, *p* = 0.046, *n* = 8; Figure 3A). We therefore propose that this red opsin “lookup table” (Figure 3A) can be pre-established in red opsin-only expressing preparations and later referenced in dual-color experiments to determine an experiment-specific maximum blue radiant exposure for crosstalk-free excitation. To quantify the improvement in blue opsin dynamic range using the lookup table approach, we compared ChR2(H134R)-evoked responses at the population-derived maximum usable 440 nm radiant exposure level (4.40 ± 1.07 mV, mean ± SEM) to peak responses with blue light exposure calibrated for individual experiments (8.88 ± 1.97 mV) (Figure 3B). We found that use of this lookup table allows an up to two-fold increase in the blue opsin response dynamic range (*p* = 0.001, *n* = 12, paired *t*-test).

## Discussion

The expansion of the optogenetic toolkit brings exciting new opportunities in neuroscience. With high-performance, spectrally shifted actuators becoming widely available, many groups have begun to newly implement multicolor optogenetic strategies, for example to stimulate converging synaptic pathways (Hooks et al., 2015; Birdsong et al., 2019; Bauer et al., 2021; Joffe et al., 2022; Rindner et al., 2022; Anisimova et al., 2023), activate intermingled cell types (Yizhar et al., 2011), or bidirectionally control neuronal populations (Han and Boyden, 2007; Zhang et al., 2007; Atallah et al., 2012; Kampasi et al., 2016; Christoffel et al., 2021; Vierock et al., 2021; Li et al., 2022). Here, we focus on synapse activation to consider how multiple experimenter-controlled variables (Figure 1B) combine to affect crosstalk. We furthermore introduce a red opsin “lookup table” approach for calibrating maximum blue light exposures on an experiment-to-experiment basis.

Indeed, stimulus irradiance and duration are often considered for their impact on crosstalk. However, our data indicates that cross-activation is a highly multidimensional issue, determined by interactions between stimulus irradiance, duration, wavelength, and additionally opsin choice. Ideally, each dimension should be properly tested when designing a dual-color optogenetic experiment. This is important as theoretical advantages for dual-color optogenetics, particularly those measured directly in opsin-expressing cells, may not translate to similar advantages in a given biological system. For example, Chronos has large photocurrents and faster kinetics than ChR2(H134R) (Klapoetke et al., 2014). However, postsynaptic responses to ChR2(H134R) activation were detected at lower irradiances (Figures 2A, C; Table 1) and durations (Figures 2B, D; Table 1) than Chronos in the AUD to PPC synaptic pathway (although, note the 440 nm stimulus used here is ∼50 nm blue-shifted from the excitation peak of Chronos). Furthermore, 440 nm stimulation consistently required lower irradiances and durations (at comparable irradiances: 405 nm, 21.06 mJ/m^2^; 440 nm, 20.50 mJ/m^2^) than 405 nm to elicit detectable responses, from both ChR2(H134R)- and Chronos-expressing fibers (Figures 2A–D; Table 1). This resulted in a narrowing of the 405 nm ideal excitation window. Thus, using near-violet stimulation to avoid red opsin activation may paradoxically promote crosstalk, as the lower sensitivity of currently available blue opsins to these wavelengths necessitates use of higher radiant exposure level stimuli. These data suggest that while favorable opsin or stimulus properties may offer theoretical advantages in dual-color applications, in-house testing is necessary to select the ideal opsin combination for any particular use case.

The ability to elicit large, crosstalk-free opsin responses is critical for experimenters. Predictably, raising stimulus irradiance or duration will increase photon exposure and thus also response magnitude (given that certain biological factors, like number of synapses, do not saturate). One limiting factor can therefore be the experimental hardware, as irradiance will be limited by the power output of the stimulus light source. In our setup, this was most noticeable in our exploration of 630 nm responses, where a comparatively weak LED was used. In contrast, stimulus duration can be increased without constraint, its impact on membrane responses limited only by the charge integration kinetics of the cell and the relatively slow process of opsin desensitization. In practice, stimulus duration may therefore offer the greatest opportunity to increase response amplitudes, given that cross-activation radiant exposure thresholds are not exceeded. Blue opsins are largely insensitive to red light (Figures 2E, F). Thus, red opsin responses can be enhanced by simply increasing stimulus radiant exposure levels, with upper light limits capped by hardware rather than crosstalk risks (however, note that cross-activation by blue stimuli may be possible by radiant exposure levels higher than tested here). In contrast, red opsins are sensitive to blue wavelengths (Figures 2A–C; Yizhar et al., 2011; Lin et al., 2013; Klapoetke et al., 2014; Oda et al., 2018; Marshel et al., 2019; Kishi et al., 2022). The need to restrict blue radiant exposure levels below the red opsin’s activation threshold can therefore limit an experimenter’s potential to elicit large blue opsin responses. Here, we propose a lookup table approach for determining the maximum crosstalk-free blue radiant exposure using a functional assessment of red opsin expression levels, thereby maximizing the dynamic range available in each individual experiment (Figure 3A). In our hands, this approach increased blue light responses up to two-fold (Figure 3B). To ensure applicability to future experiments, the lookup table should encompass a large range of expression levels in each pathway or cell population. While light-evoked responses often scale with the observed brightness of the fluorescent tag carried by the opsin, this relationship can be construct specific and, in some cases, fluorescence signal from the tag may not ensure the presence of an evoked response (Klapoetke et al., 2014). We therefore propose that directly measured opsin responses are a more reliable predictors of crosstalk thresholds when constructing this lookup table.

In synapse activation experiments, crosstalk can be measured in opsin-expressing cells or downstream in postsynaptic neurons. We agree with previous studies (Birdsong et al., 2019; Xia et al., 2020; Joffe et al., 2022) that downstream measurements better capture the functional implication of cross-activation. Nonetheless, it is likely that ChrimsonR photocurrents were activated at lower blue light intensities and durations than discernible from postsynaptic responses alone. However, this stimulus may either not activate enough ChrimsonR to trigger action potential firing in the afferents or engage enough synapses to produce a detectable response in the recorded postsynaptic neuron. Additionally, while the lookup table approach can improve blue opsin response ranges, it may overestimate crosstalk-free light levels for adjacent cells more strongly innervated by opsin expressing afferents. It is important to remember that crosstalk is subject to filtering by the biological system studied. In extreme cases, indirect measures including behavioral (Schild and Glauser, 2015) or paired-pulse response characteristics (Christoffel et al., 2021) can also been used as evidence against appreciable crosstalk, although opsin cross-activation almost certainly occurred. It is the experimenter’s responsibility to consider subthreshold effects of such cross-activation and determine their potentially confounding influence on the process studied. As in any experiment, there is no substitute for high-quality controls for ensuring the validity of results. Whenever possible, swapping opsins between cell populations is one such necessary control.

Cross-activation concerns are not specific to excitatory actuator pairs. Other optogenetic tool combinations are equally subject to crosstalk, including excitatory with inhibitory actuators (Han and Boyden, 2007; Zhang et al., 2007; Atallah et al., 2012; Kampasi et al., 2016; Christoffel et al., 2021; Vierock et al., 2021; Li et al., 2022), actuators with sensors (Lim et al., 2012; Rickgauer et al., 2014; Packer et al., 2015; Carrillo-Reid et al., 2016; Yang et al., 2018; Seidenthal et al., 2023), and sensors with sensors (Zhao et al., 2011; Akerboom et al., 2013; Li et al., 2013; Han et al., 2019). Multicolor sensitivity is also a core feature of bistable step-function opsins (Berndt et al., 2009; Yizhar et al., 2011; Wietek et al., 2017; Gong et al., 2020), where an additional wavelength-to-response relationship must be carefully considered. Efforts to produce optogenetic tools with decreased blue light sensitivity and further red-shifted peak activation are ongoing (Mermet-Joret et al., 2021; Kishi et al., 2022). Parallel modifications to blue-activated tools may also decrease crosstalk risk. High-photocurrent, high-sensitivity blue opsins, such as ChRger2, could further reduce the necessary blue radiant exposure to stimulate neurons (Bedbrook et al., 2019), thus reducing crosstalk risk. Opsins with fast activation kinetics, such as Chronos and ChroME (Mardinly et al., 2018), have also been successfully applied in multicolor experiments. A third avenue that saw limited success was to further blue-shift action spectra. TsChR, a channelrhodopsin discovered in *T. striata*, has an excitation peak at 430 nm and is the most blue-shifted channelrhodopsin known to date (Klapoetke et al., 2014). Membrane trafficking of TsChR was further optimized to generate eTsChR (Farhi et al., 2019). While promising, the efficacy of eTsChR in multicolor applications has yet to be verified. The continued spectral and kinetic expansion of the optogenetic toolkit, combined with experimental strategies for minimizing crosstalk, will ultimately broaden the adoption of dual-color approaches for studying brain function.

## Methods

### Animals

Wild-type C57BL/6 mice of both sexes were used for all experiments. Mice were bred and maintained with *ad libitum* access to food and water on 12-h light/dark cycles in University of California, Irvine vivarium facilities. All mice were housed and used in accordance with the NIH guidelines on the care and use of laboratory animals.

### Optogenetic construct expression

To express various optogenetic constructs, we transcranially injected either AAV2.9-hSyn-hChR2(H134R)-EYFP (titer: 3.6 × 10^12^, Addgene: 26973-AAV9) (Zhang et al., 2010), AAV2.9-hSyn-ChrimsonR-tdTomato (titer: 2.9 × 10^12^, Addgene: 59171-AAV9) (Klapoetke et al., 2014), or AAV2.9-Syn-Chronos-GFP.WPRE.bGH (titer: 1.6 × 10^12^, originally obtained from the Penn Vector Core, now available from Addgene: 59170) (Klapoetke et al., 2014). Injections were targeted to auditory cortex (AUD, coordinates from bregma: anterior-posterior −2.8 mm, medial-lateral 4.1 mm, dorsal-ventral 0.8 mm) under isoflurane anesthesia between postnatal day (p) 28–40. Opsin constructs (150 nL) were injected at a rate of 25 nl/minute and allowed to express for (in days): ChR2(H134R), 32.2 ± 5; ChrimsonR, 29.9 ± 2.4; Chronos, 28.0 ± 7.2; mean ± standard deviation. Opsin expression was visually inspected at the beginning of all physiology experiments via band-pass illumination with a X-Cite mercury lamp (Excelitas Technologies, Waltham, MA, United States). Slices were discarded if opsin-expressing fibers were not visually apparent.

### Electrophysiology

Electrophysiology recordings were performed in 400 μm thick coronal brain slices prepared on a vibrating microtome (smz7000-2, Campden Instruments, Lafayette, IN, United States) cut to contain the PPC (approximate coordinates from bregma: anterior-posterior: 2.0 mm, medial-lateral: 1.3–1.8 mm). After cutting, slices were maintained at 32°C for 15 min in solution comprised of (in mM): 110 choline, 25 NaHCO_3_, 1.25 NaH_2_PO_4_, 3 KCl, 7 MgCl_2_, 0.5 CaCl_2_, 10 glucose, 11.6 sodium ascorbate, and 3.1 sodium pyruvate, bubbled with carbogen gas (95% O_2_, 5% CO_2_). Slices were then transferred to room-temperature ACSF comprised of (in mM): 126 NaCl, 25 NaHCO_3_, 1.25 NaH_2_PO_4_, 3 KCl, 1 MgCl_2_, 2 CaCl_2_, and 10 glucose, and allowed to recover for a minimum of 20 min before recording.

Whole-cell recordings were performed in a submersion-type recording chamber mounted on an Olympus, Tokyo, Japan BX61-WI microscope. The extracellular bath solution consisted of oxygenated ACSF maintained at close to physiological temperature (32–34°C). Recording glass micropipettes were pulled on a P-1000 puller (Sutter Instrument, Novato, CA, United States) to have pipette resistances of 2–4 MΩ. Intracellular solution contained (in mM): 135 KMeSO_3_, 10 HEPES, 4 MgCl_2_, Na_2_ATP, 0.4 NaGTP, and 10 sodium creatine phosphate, adjusted to pH 7.3 with KOH. Signals were amplified on a MultiClamp 700B amplifier (Molecular Devices, San Jose, CA, United States) and digitized on National Instruments DAQ boards. Data were sampled at 10 kHz, filtered at 4 kHz, and acquired using WaveSurfer (HHMI Janelia Research Campus). Recordings were targeted to pyramidal cells in layer 5 (depth from pia: 0.5–0.65 mm), identified by their characteristic wide somatic morphology and non-fast-spiking firing patterns. Cells were held at ∼−75 mV, and series resistance constantly monitored for changes greater than 20% at which point cells were discarded. All analysis routines were custom written in Python 3.7.

### Multi-color optogenetic stimulation

Multi-color excitation light was delivered through a 40x water immersion objective lens (Olympus, Tokyo, Japan). 405 nm, 440 nm, and 630 nm high speed LEDs (Sutter Instrument, Novato, CA, United States) were integrated into the light path using an optical beam combiner (Lambda OBC, Sutter Instrument, Novato, CA, United States) mounted directly to the microscope. We used a reverse mounting order such that higher power LEDs were mounted further from the OBC output. Stimulus properties (irradiance, duration) were controlled using WaveSurfer with a sampling rate of 100 kHz. All LED irradiances were tested using a stimulus duration of 100 μs and measured at the microscope objective with a PM100D power meter and S121C sensor (Thorlabs, Newton, NJ, United States). All durations were tested with a stimulus irradiance of (in mW/cm^2^): 21.06 (405 nm), 20.50 (440 nm), or 8.23 (630 nm).

### Experimental design and statistical analysis

On occasion, stimulus parameters were sufficient to evoke action potentials in recorded neurons. In these instances, EPSP amplitudes were fixed at 25 mV. The PPC and AUD are reciprocally connected (Zingg et al., 2014), raising the risk of retrograde opsin expression in PPC contributing to evoked responses. However, opsin-expressing PPC cells were never observed, excluding this possibility. Non-zero postsynaptic responses were determined by comparing the maximum post-stimulus membrane potential to the peak-to-peak noise estimate taken pre-stimulus using paired *t*-tests performed in Python 3.7. Stimulus parameters were tested through 10 repeated measurements in each cell. Each repeat was considered a single experimental replicate (*n*) when determining single cell response minimums. Responses from a given neuron were averaged to form a single experimental replicate when determining population response minimums. No more than two cells were recorded from the same animal for a given condition.

## Data availability statement

The raw data supporting the conclusions of this article will be made available by the authors, without undue reservation.

## Ethics statement

This animal study was reviewed and approved by University of California, Irvine IACUC, AUP-20-076.

## Author contributions

DR performed all recordings, analyzed the data, and wrote the manuscript. GL provided experimental resources and editorial comments. Both authors contributed to the experimental design and approved the submitted version.
